# Can sports participation be a protective factor against suicide-related outcomes in adolescents: a systematic review

**DOI:** 10.3389/fpsyg.2024.1341795

**Published:** 2024-06-04

**Authors:** Meilin Huo, Zhen Yang, Li Yang, Sitong Chen

**Affiliations:** ^1^Department of Physical Education, Huaide College of Changzhou University, Changzhou, China; ^2^Department of Movement Sciences, KU Leuven, Leuven, Belgium; ^3^School of Physical Education, South China University of Technology, Guangzhou, China; ^4^Centre for Mental Health, Shenzhen University, Shenzhen, China

**Keywords:** sports participation, protective factor, suicide-related outcome, adolescent, systematic review

## Abstract

**Background:**

Suicide-related outcomes among adolescents have become a serious public health problem worldwide. Emerging evidence suggests that sports participation may interact with suicide-related outcomes. The objective of this systematic review is to systematically review and summarize the association between sports participation and suicide-related outcomes among adolescents.

**Design:**

A systematic review according to PRISMA Statement (International Platform of Registered Systematic Review and Meta-Analysis Protocols registration: INPLASY202330072) https://inplasy.com/inplasy-2023-3-0072/.

**Methods:**

The literature search was conducted in three electronic databases: Web of Science, PubMed, and EBSCOhost.

**Results:**

A total of 16 studies (12 cross-sectional studies, 4 prospective studies) met the inclusion criteria were evaluated. Strong consistent evidence was found for the negative association between suicidal ideation, suicide attempts, and sports participation among adolescents. There was insufficient evidence to support consistency in the association between sports participation and suicide plans, and no evidence regarding gender differences between sports participation and these suicide-related outcomes. Furthermore, there was heterogeneity in the measures of sports participation and suicide-related outcomes across the included studies.

**Conclusion:**

Evidence suggests that more sports participations are associated with lower suicidal ideation and suicide attempts in adolescents. However, the directionality of the observed associations should be examined based on more high-quality longitudinal studies in the future.

## 1 Introduction

Suicide is a serious global public health problem, with 817,000 people terminating their lives each year (Naghavi, 2019; World Health Organization, 2021). The World Health Organization estimates that suicide is the fourth leading cause of death among people aged 15–29 years (World Health Organization, 2022), and more than 60,000 children and adolescents die each year as a result of suicide (Naghavi, 2019). Suicide rates are high among adolescents in Eastern and Central Europe, the high-income Asia-Pacific region, and the high-income North America region (Naghavi, 2019). Regarding gender differences, suicide rates are higher among males than females, except in the 15–19 age group (Naghavi, 2019). Despite the fact that suicide rates increase with age, it is the second most common cause of death among adolescents in Europe (Bilsen, 2018). While suicide is not even one of the top 10 common causes of death among older people. Previous studies indicated that age standardized mortality rates declined significantly between 1990 and 2016 in a significant number of countries, notably China, Denmark, and the Philippines (Naghavi, 2019). Although the suicide rate has declined significantly since 1990, as a result of successful public health actions, increases in the prevalence of suicide among adolescents has been observed in some regions due to the COVID-19 pandemic (Bersia et al., 2022; Goto et al., 2022).

In addition to death by suicide, suicidal ideation, suicide plans, and suicide attempts are modifiable suicide-related outcomes (O’Connor et al., 2013). Those suicide-related outcomes, especially among adolescents, is complex and results from the interaction of social, psychological, environmental and genetic risk factors (Wilcox et al., 2010). Suicidal ideation (or suicidal thoughts), which is defined as any thoughts about ending one’s own life, is considered the first step on the road to suicide (Kachur et al., 1995; Turecki et al., 2019). As suicidal ideation becomes more frequent and planned, the risk of taking actual suicidal action increases (Turecki et al., 2019). Suicide attempts, which is defined as an action of self-harm with the intention of presumed or actual death, may or may not result in death (Turecki et al., 2019). In the recent meta-analysis, the prevalence of suicidal ideation among 6- to 21-year-olds people varied by region, ranging from 14.3 to 22.6%, while the prevalence of suicide attempts ranged from 4.6 to 16.9% (van Meter et al., 2023). Another meta-analysis of 686,672 children and adolescents showed that the lifetime prevalence of suicidal ideation, plans, and attempts was 18.0, 9.9, and 6.0%, respectively (Lim et al., 2019). Given the complexity of suicide the actual number of deaths by suicide may be greatly underestimated due to unrecognized or misattributed (De Leo, 2015). Furthermore, the frequency of suicide-related outcomes is much higher, being estimated to be 10–20 times higher than the frequency of the death by suicide (Bilsen, 2018).

Suicide is not only an individual tragedy but also affects family, peers, and communities (Cerel et al., 2019). Furthermore, suicide and suicide-related outcomes can also be a heavy financial loss. It is estimated that suicide and suicide attempts cost the United States $58.4 billion each year (Shepard et al., 2016), and the average cost of suicide for adolescents in the most developed countries is more than $ 800,000 (Doran and Kinchin, 2020). In developing countries, the economic costs of suicide and suicide-related outcomes are even more severe, not only due to the associated medical burden, but also in terms of future lost productivity. Therefore, timely, evidence-based, and cost-effective interventions are required to address this global public health challenge (World Health Organization, 2021).

Previous research has identified genetic, biological, psychological, and social factors as risk factors for suicide in children and adolescents (Bilsen, 2018). While healthy lifestyle, such as sports participation, is identified to be protective factors for suicide and suicide-related outcomes among adolescents (Grasdalsmoen et al., 2020; Wasserman et al., 2021). This promising behavior is defined as purposeful active participation in sports related physical activities performed during leisure-time (Scheerder et al., 2005). Previous research showed that the prevalence of suicide-related outcomes was lower in high school and college students who participated in sports than those who did not (Lester, 1995; Brown and Blanton, 2002; Sabo et al., 2005; Brown et al., 2007; Babiss and Gangwisch, 2009; Taliaferro et al., 2011; Cho, 2014; Hu and Tang, 2022). Studies in the United States, Korea, and Canada reported that high school students who participated in school sports teams had lower odds of suicidal ideation (Dugas et al., 2012; Southerland et al., 2016; Park et al., 2020). Furthermore, among high school and college students in the United States, a lower incidence of suicide plans was found among those with higher sports participation compared to those with lower sports participation (Brown and Blanton, 2002; Sabo et al., 2005; Xiao et al., 2019). Meanwhile, participation in sports teams has also been reported to be protective factors for the suicide plans among secondary school students (Southerland et al., 2016). Previous studies suggested that sports participation is negatively associated with the incidence of suicide attempts among high school students (Brown and Blanton, 2002; Brown et al., 2007; Cho, 2014; Grasdalsmoen et al., 2020; Hu and Tang, 2022). Furthermore, suicide attempts were higher among high school and college students who did not participate in sports teams than among those who did (Southerland et al., 2016; Li et al., 2021).

Despite the fact that numerous studies have explored the association between sports participation and suicide-related outcomes in adolescent populations, there is no systematic review to summarize the extant evidence. Vancampfort et al. systematically reviewed the association between physical activity and suicidal ideation (Vancampfort et al., 2018a). However, Shull et al. considered sports participation is a subset of physical activity (Shull et al., 2020). Meanwhile, other research proposed that sports participation sometimes involves sedentary behavior and light physical activity (Leek et al., 2011; Guagliano et al., 2013). Therefore, a distinction needs to be performed between research on physical activity and sports participation. Although Zuckerman et al. systematically reviewed studies of team sports participation and suicidal ideation in young athletes, other suicide-related outcomes were ignored, and the participants were young athletes under the age of 25, the findings of these results are difficult to generalize to general adolescents (Zuckerman et al., 2021).

Accordingly, this systematic review aims to summarize and evaluate the association between sports participation and suicide-related outcomes in adolescents in observational studies and conduct gender subgroup analyses because of differences in suicide risk by gender.

## 2 Materials and methods

This present systematic review was designed and conducted according to the Preferred Reporting Items for Systematic Reviews and Meta-Analyses (PRISMA) guideline (Page et al., 2021). A systematic review protocol had been registered in the International Platform of Registered Systematic Review and Meta-Analysis Protocols (INPLASY202330072).

### 2.1 Search criteria and study selection

Electronic databases including Web of Science, PubMed, and EBSCOhost were applied for the data search from inception to November 2022 by two individual authors (MH and ZY). The keywords, search strategies, and Boolean logic operators were performed for retrieval. Three search fields focusing on adolescent population, sports participation, and suicide-related outcomes were connected using ‘AND’ and the full description of search strategies was available in the supplementary materials. After removing duplicates, the tittles and abstracts of all potentially eligible articles were screened, whose full text were then considered based on the eligibility criteria. In the event of a contradiction, the third author (LY) made the final decision.

### 2.2 Eligibility criteria

This present systematic review focused on the association between sports participation and suicide-related outcomes among adolescents. Therefore, studies were included in this systematic review if they met the following criteria: (1) observationally designed and published in peer-reviewed journals; (2) conducted to explore the association between sports participation and suicide-related outcomes among adolescents (aged 12–18 years), and (3) performed to report any type of effect size [e.g., odds ratio (OR) correlations, *t*-tests, and Chi-squared tests]. Variables related to sports participation such as physical activity, aerobic exercise were excluded because sports participation is a complex concept that has been reported to also involve sedentary behavior and light physical activity (Leek et al., 2011; Guagliano et al., 2013). Furthermore, non-English publications, case reports, expert opinions, comments, conference abstracts, etc. were excluded.

### 2.3 Data extraction and quality assessment

Data from the included studies were extracted and checked independently by two authors (MH and ZY) and re-validated by the third author (LY). Data extracted included study design (cross-sectional, prospective cohort study, longitudinal study), first author’s name, year of publication, participants’ sample size, gender ratio, and age (mean or range), exposure variables, outcome variables, and most adjusted odds ratios (ORs) with 95% confidence intervals (95%CI) of associations between sports participation and suicide-related outcomes.

Studies included in this systematic review were assessed for quality by the Strengthening the Reporting Guidelines for Observational Studies in Epidemiology (STROBE) (Von Elm et al., 2014), a 22-item reporting guideline for observational studies, which contains the following six instructions to measure the quality of included studies: (1) Study Aim; (2) Issues Explored; (3) Study Design; (4) Sample Size; (5) Participant Characteristics; and (6) Results. Each study was rated based on its summary score in the six instructions in STROBE, in which 0–2 represents poor, 3–4 represents good, and 5–6 represents excellent.

### 2.4 Coding and data synthesis

In accordance with previous methodological approaches (Sallis et al., 2000), codes reporting the association between sports participation and suicide-related outcomes in observational studies were expressed as a percentage. This percentage represents the proportion of studies that support the association between sports participation and suicide-related outcomes. Referring to previous relevant studies (Vancampfort et al., 2012, 2018a), this association was defined as ‘non-existing’ if only 0–33% of the studies supported the association. If 34–59% of studies supported the association, then the association was considered as ‘unclear’. When 60–100% of the studies support the association, then the association was defined as ‘existing’. Furthermore, the association was refereed as ‘consistent in literature’ if it was reported in four or more studies (Vancampfort et al., 2012, 2018b).

## 3 Results

### 3.1 Study selection

**Figure 1:**
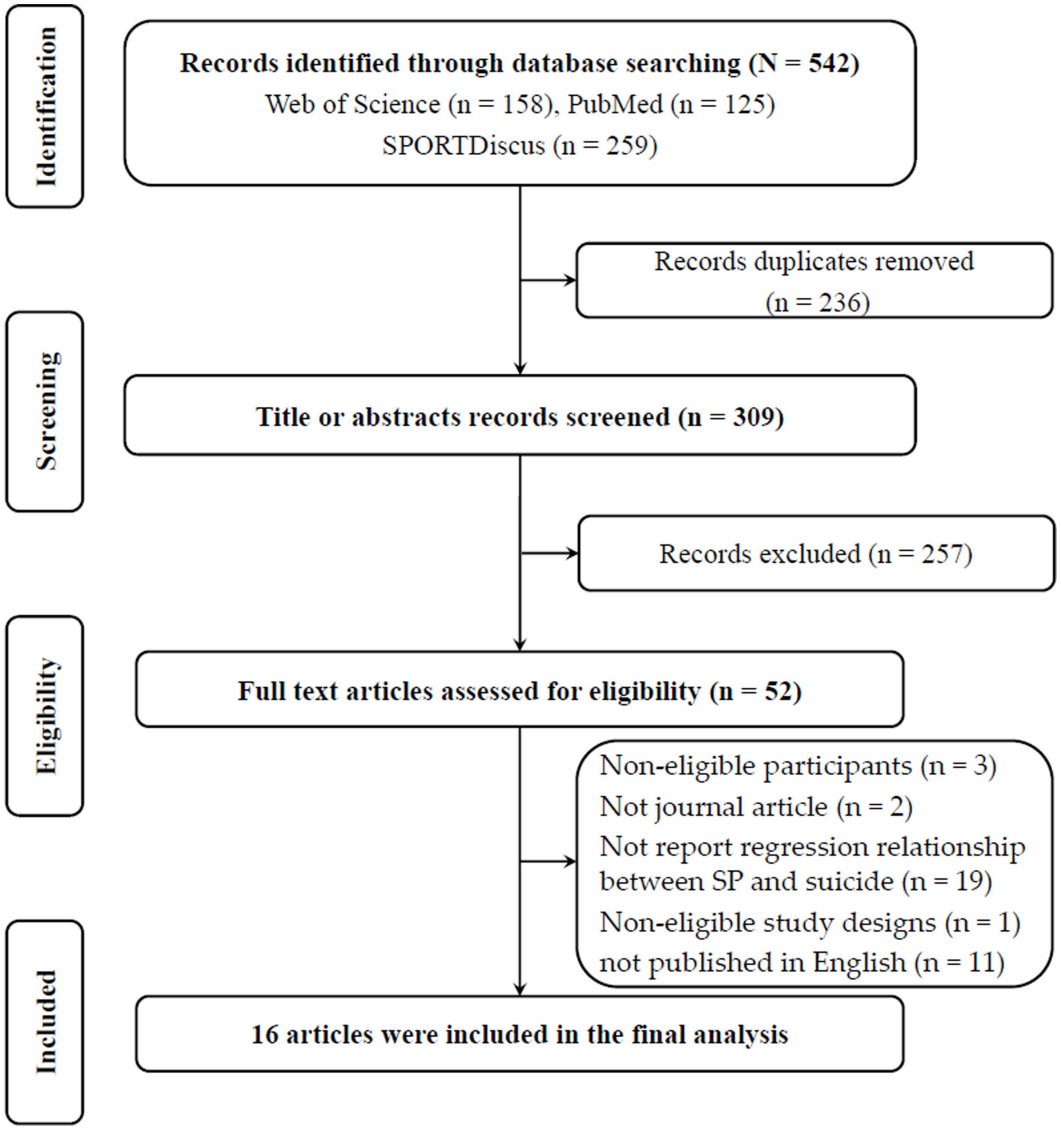
Preferred Reporting Items in Systematic Reviews and Meta-analyses (PRISMA) flow diagram for selecting the studies.

A total of 542 articles were initially retrieved from the electronic database and after excluding 236 duplicate records, 309 articles were considered at the title and abstract level. A total of 52 articles were reviewed for full text after 257 irrelevant records were dispatched. Nineteen articles were excluded due to the regression association between the sports participation and suicide-related outcomes was not reported, 11 articles were excluded due to not being published in English, 3 articles were excluded as non-eligible participants, 2 articles were excluded as not journal articles, and 1 article was excluded as non-eligible study design. Sixteen articles were eventually included in the analysis, the Figure 1 presented the flow chart of the study selection.

### 3.2 Participant and study characteristics

The final total sample size of the included studies was 337,283 independent participants, with a median sample size of 21,080 for each study. Among 16 included studies, there were 12 cross-sectional studies (Unger, 1997; Brown et al., 2007; Taliaferro et al., 2008; Babiss and Gangwisch, 2009; West et al., 2010; Taliaferro et al., 2011; Mata et al., 2012; Gunn III and Lester, 2014; Southerland et al., 2016; Lester, 2017; Rodelli et al., 2018; Michael et al., 2020; Li et al., 2021), 3 prospective cohort studies (Dugas et al., 2012; Iverson et al., 2021; Iverson and Terry, 2022), and 1 longitudinal study (Taliaferro et al., 2011). The sample sizes of the included studies ranged from 739 to 152,858, with a balanced gender distribution in the majority of studies. Only one study did not report a gender ratio (West et al., 2010), and two prospective longitudinal studies in which the participants were all male (Iverson et al., 2021; Iverson and Terry, 2022). All of the studies were conducted in high-income countries, with the United States accounting for the vast majority (87.50%), and Canada and Belgium each had one (Dugas et al., 2012; Rodelli et al., 2018). The exposure variable in the majority of studies was sports participation, with two studies specific to football participation (Iverson et al., 2021; Iverson and Terry, 2022). In terms of suicide-related outcomes, 93.75% studies measured suicidal ideation (or suicidal thoughts), 25% studies assessed suicide plans, while 68.75% studies measured suicide attempts. In terms of measuring sports participation, all studies used self-reported instruments which had unknown reliability and validity in investigating sports participation. To measure suicide-related outcomes, all studies used self-reported instruments. None of the studies used an objective measurement tool for measuring sports participation or suicide-related outcomes. The most commonly used statistical method to assess the association between sports participation and suicide-related outcomes was logistic regression analysis (75.00%). The mean STROBE score of 16 studies was 5.125 (range = 4–6), while 12 and 4 studies had excellent and good methodological quality, respectively. Table 1 summarized the characteristics of included studies, while results of quality assessment of included studies was in Table 2.

**Table 1:** Study characteristics of the included studies of this review.

	First Author	Publish Year	Study Design	Age (mean or range)	Sample Size	Male %	Exposures	Outcomes	Results
1	Unger	1997	C	12–18	10,506	48.8	Team sports participation	1.Thought about suicide 2.Planned suicide 3.Attempted suicide 4.Multiple suicide attempt	1.Compared with those not reporting any physical activity, physical activity plus team sports was associated with lower risk of thinking about suicide (OR = 0.48* - 0.58*), planning suicide (OR = 0.47* - 0.58*), attempting suicide (OR = 0.40*- 0.50*) and of multiple suicide attempts (OR = 0.18* - 0.42*) in boys. 2.Physical activity of at least six times per week plus team sports was associated with lower risk of planning suicide (OR = 0.43*) in girls, but physical activity plus team sports was associated with higher risk of thinking about suicide (OR = 1.25*-1.33*) and attempting suicide (OR = 1.50*) in girls.
2	Brown	2007	C	14–18	10,530	48.6	Sports team participation	1.Suicidal ideation 2.Suicide attempts	1.Compared with sports team nonparticipants, the odds of suicidal ideation were lower among boys reporting sports team participation (AOR = 0.65; 95% CI = 0.48, 0.86). The odds of suicide attempts were also lower among sports team participants (AOR = 0.61; 95% CI = 0.40, 0.93). 2.The odds of suicide attempts were lower for sports team participants compared with nonparticipants (AOR = 0.73; 95% CI = 0.57, 0.94).
3	Taliaferro	2008	C	12–18	13,857	48.1	Sports participation	1.Thought about suicide 2.Planned suicide 3.Attempted suicide 4.Multiple suicide attempt	1.Male athletes were less likely to think (OR = 0.71*), plan (OR = 0.76*), or attempt (OR = 0.70*) suicide. Highly involved male athletes had reduced odds of thinking (OR = 0.66*) or planning (OR = 0.68*) suicide than nonathletes. 2.Female athletes demonstrated reduced risk of thinking suicide (OR = 0.80*), planning suicide (OR = 0.79*), and attempting suicide multiple times (OR = 0.76*). Compared to nonathletes, highly involved female athletes were less likely to think suicide (OR = 0.79*).
4	Babiss	2009	C	11–21	14,594	51.2	Sports participation	Suicidal ideation	1.As sports participation increases, the odds of having suicidal ideation decreases by 12% (OR: 0.88; 95% CI: 0.83 0.93) after controlling for sex, age, race/ethnicity, public assistance, and physical limitations. 2.Substance abuse, body weight, and exercise did not mediate these associations. 3.Consistent with self-esteem and social support acting as mediators of these relationships, the inclusion of these variables in the multivariate models attenuated the associations for suicidal ideation (OR: 0.93; 95% CI: 0.88 0.99).
5	West	2010	C	Grade 9–12	14,041	/	Sports involvement	Suicide attempt	Among boys, sports involvement (AOR = 1.52*) were significantly associated with suicide attempts, but not in girls.
6	Taliaferro	2011	L	time 1: 11–15 time 2: 15–18	739	44.1	Sports participation	1.Suicidal ideation 2.Suicide attempts	1.Compared to non-participants, youth involved in sports in both middle and high school had lower odds of suicidal ideation during high school. 2.Youth who discontinued sports after middle school had higher odds of attempting suicide during high school than non-participants.
7	Dugas	2012	PC	time 1: 12–13 time 21: 20.4 ± 0.7	877	46	Participation in sports teams at school	Suicidal ideation	In grade 8, participation in sports teams in and (or) outside of school protected against suicidal ideation (OR = 0.6*; 95% CI 0.4 to 0.8).
8	Mata	2012	C	11–18	13,977	48.1	Sport	Suicidal ideation Suicide attempts	1.Early adolescent sports participants were less likely to think about suicide compared with early adolescent sports nonparticipants (*β* = 19*). 2.School belongingness may play a role in understanding the association between extracurricular activity involvement and adolescent suicide risk.
9	Gunn	2014	C	15.6 ± 1.8	6,485	48.4	Sports participation	Suicidal ideation	1.The full model with all predictors was able to predict suicidal ideation (*χ*2 = 26.57, df = 12*). For boys, basketball, and soccer were protective (OR = 0.63* and 0.51*, respectively). However, boys engaged in cheerleading/dance were more likely to report suicidal ideation (OR = 4.10*). 2.Baseball/softball was associated with a lower incidence of suicidal ideation (OR = 0.60*), while wrestling was associated with an increased incidence of suicidal ideation (OR = 4.02*) in girls. 3.For Black, Asian, Hispanic, and Native American adolescents, only seven phi coefficients were statistically significant, and participation in all of these increased the incidence of suicidal ideation: swimming for Black girls, baseball, and volleyball for Asian males, football/soccer for Asian females, cheerleading/dance for Hispanic males, field hockey for Hispanic females, and track for Native American females.
10	Southerland	2016	C	12.81 ± 1.04	65,182	50.58	Sports team engagement	1.Suicidal thoughts 2.Suicide plan 3.Suicide attempts	Sports team engagement was significantly associated with suicidal thoughts (OR = 0.65, 95% CI = 0.62–0.69), plan (OR = 0.66, 95% CI = 0.62–0.70), and attempts (OR = 0.70, 95% CI = 0.65–0.75) even after controlling for other important variables.
11	Lester	2017	C	13–18	152,858	48.8	Participation in sports teams	1.Suicidal ideation 2.Suicide attempts	1.Participation in sports was associated with students reporting suicidal ideation less often in the prior year (16.9% versus 21.1%) and less often reporting suicide attempts (7.6% versus 9.8%). 2.For African American, Hispanic American and Asian-American girls, participation in sports might be a risk factor for suicidal ideation and suicide attempts. 3.For boys from minority ethnic groups, participation in sport was not associated with suicidal behavior.
12	Rodelli	2018	C	12–18	1,037	50	1.Participates in leisure time sports 2.Participates in outdoor leisure time sports	Suicidal ideation	While more frequent cyberbullying by standing was associated with higher suicidal ideation, adolescents who participated in sports had higher odds of suicidal ideation at less frequent by standing than those who did not participate in sports (OR = 1.26, 95% CI = 9.85–1.87).
13	Michael	2020	C	Grade 9–12	14,765	49.3	Did not play on at least one sports team	1.Seriously considered attempting suicide 2.Attempted suicide	Playing on at least one sport team were not significantly associated with suicide attempts.
14	Iverson	2021	PC	time 1: 14.9 ± 1.8 time 4: 29.1 ± 1.8	2,353	100	Football participation	1.Suicidal ideation 2.Suicide attempts	1.Men who played high school football, compared with those who did not, reported similar rates of lifetime diagnosis of suicidal ideation in the past year [5.1% vs. 5.9%; *χ*2(1) = 50.408, OR = 0.857, 95% CI = 0.533–1.377]. 2.Those who played football reported similar rates of suicidal ideation in the past year when they were in their early 20s. [6.3% vs. 6.3%; *χ*2(1) = 0.005, OR = 0.983, 95% CI = 0.603–1.603].
15	Li	2021	C	14–18	13,677	48.6	Sports team participation	1.Considered attempting suicide 2.Planned suicide 3.Attempted suicide	Participants who did not engage in any sport team were more likely to report think (OR = 1.50*), plan (OR = 1.31*), and attempt (OR = 1.49*) suicide.
16	Iverson	2022	PC	time 1: median age = 15 time 4: median age = 38	1,805	100	Football participation	Suicidal ideation	When comparing men who played high school football to those who did not, there were no differences in the proportions of the sample who had a suicidal ideation [6.0 vs. 7.0%; *χ*2(1) = 0.49, OR = 0.84, 95% CI = 0.51–1.38].

C, cross-sectional study; PC, prospective cohort study; L, longitudinal study.

**Table 2:** Results of quality assessment of the included studies in this review.

	Author (Year)	Study Aim	Study question	Study Design	Sample Size	Participant Characteristics	Results	Total
1	Babiss and Gangwisch (2009)	1	0	0	1	1	1	4
2	Brown et al. (2007)	1	1	1	1	1	1	6
3	Dugas et al. (2012)	1	0	1	1	1	1	5
4	Mata et al. (2012)	1	1	0	1	1	1	5
5	Taliaferro et al. (2008)	0	0	1	1	1	1	4
6	Taliaferro et al. (2011)	1	0	1	1	1	1	5
7	Unger (1997)	1	0	1	1	1	1	5
8	West et al. (2010)	1	1	1	1	1	1	6
9	Gunn III and Lester (2014)	1	0	0	1	1	1	4
10	Southerland et al. (2016)	1	1	1	1	1	1	6
11	Lester (2017)	1	0	0	1	1	1	4
12	Rodelli et al. (2018)	1	1	1	1	1	1	6
13	Michael et al. (2020)	1	1	1	1	1	1	6
14	Iverson et al. (2021)	1	0	1	1	1	1	5
15	Li et al. (2021)	1	1	1	1	1	1	6
16	Iverson and Terry (2022)	1	0	1	1	1	1	5

### 3.3 Association between sports participation and suicide-related outcomes.

Table 3 summarized the associations between sports participation and suicide-related outcomes among adolescents. In terms of suicidal ideation, 78% (7/9) of studies supported a negative association between sports participation and suicidal ideation (i.e., higher sport participation was associated with lower suicidal ideation and vice versa). In the gender subgroup analysis, only 33% (2/6) and 43% (3/7) of the studies supported this association in males and females respectively, so it is presently unclear whether there is a consistent association in girls or boys. In terms of suicide plans, the association is inconsistent as there were fewer than four studies available. Furthermore, 67% (4/6) of the studies supported a negative association between sports participation and suicide attempts. In the gender subgroup analysis, only 14% (1/7) and 43% (3/7) of studies supported the association in males and females respectively, so it is currently unclear whether there is a consistent association in boys and girls.

**Table 3:** Synthesiszed results for the associations between sports participation and suicide-reltead outcomes.

	Ass.	Girl	Boy	Total
		No.	No.	No.
Suicidal ideation/ Suicidal thoughts	-*	Taliaferro et al. (2008); Lester (2017)	Unger (1997); Taliaferro et al. (2008); Lester, 2017	Babiss and Gangwisch (2009); Taliaferro et al. (2011); Dugas et al. (2012); Mata et al. (2012); Southerland et al. (2016);Lester (2017); Li et al. (2021)
	+*	Unger (1997)		Rodelli et al. (2018)
	-	Brown et al. (2007)	Brown et al. (2007); Iverson et al. (2021)	Iverson and Terry (2022)
	+	Michael et al. (2020)	Michael et al. (2020)	
	Undetermined	Gunn III and Lester (2014)	Gunn III and Lester (2014)	
n^-*^/N (%)		2/6 (33%)	3/7 (43%)	7/9 (78%)
Ass.		?	?	-*
Suicide plans	-*	Unger (1997); Taliaferro et al. (2008)	Unger (1997); Taliaferro et al. (2008)	Southerland et al. (2016); Li et al. (2021)
n^-*^/N (%)		(n ≤ 4)	(n ≤ 4)	(n ≤ 4)
Ass.		?	?	?
Suicide attempts	-*	Lester (2017)	Unger (1997); Taliaferro et al. (2008); Lester (2017)	Mata et al. (2012); Southerland et al. (2016); Lester (2017); Li et al. (2021)
	+*	Unger (1997)	West et al. (2010)	
	-	Brown et al. (2007); Taliaferro et al. (2008); West et al. (2010); Iverson et al. (2021)	Brown et al. (2007); Iverson et al., 2021	Taliaferro et al., 2011
	+	Michael et al. (2020)	Michael et al. (2020)	West et al. (2010)
n^-*^/N (%)		1/7 (14%)	3/7 (43%)	4/6 (67%)
Ass.		?	?	-*

No., reference number; Ass., association; +, positive association; −, negative association; *, significant associations;?, indeterminate/inconsistent association, indicates 34–59% of studies and less than four studies supporting a significant association.

## 4 Discussion

Systematically evaluating the association between sports participation and suicide-related outcomes among adolescents is considered of great public health significance because this information can inform interventions to reduce suicide-related behaviors in youth populations (Vancampfort et al., 2018a). To our knowledge, this present systematic review is one of the first studies to explore the association between sports participation and suicide-related outcomes in adolescents. While a large number of cross-sectional studies were included to determine this association, there was a lack of longitudinal studies examining the direction of the association between sports participation and suicide-related outcomes among adolescents. Based on good- to excellent-quality cross-sectional and longitudinal studies, the findings of this systematic review indicate evidence for a negative association between sports participation and suicidal ideation and suicide attempts among adolescents. However, it is currently unclear whether there is a consistent association between sports participation and suicide plans among adolescents. In gender-stratified subgroup analyses, it is uncertain whether the association between sports participation and suicidal ideation, suicide plans, and suicide attempts is consistent across males and females.

Previous systematic reviews and the current study differed in the population examined, the exposure variables, and outcome variables, thus drew inconsistent conclusions (Vancampfort et al., 2018a; Zuckerman et al., 2021). Our study finds that sports participation is negatively associated with suicidal ideation and suicide attempts. Zuckerman et al. reported that all three included studies supported that participation in team sports was associated with lower suicidal ideation (Zuckerman et al., 2021). However, Vancampfort et al. (2018a) study indicated that 7 of 14 studies in adolescent populations found a negative association between physical activity and suicidal ideation, but it was uncertain whether there was a consistent association. While the association between sports participation and suicide attempts among adolescents has not been reported in previous systematic reviews comprising observational studies. Fabiano et al. reported that more physical activity was associated with fewer suicide attempts in clinical populations (Fabiano et al., 2024). Evidence from randomized controlled trials also demonstrates that exercise reduces suicide attempts (Fabiano et al., 2023). However, it is currently unclear whether there is a consistent association between sports participation and suicide plans among adolescents. Meanwhile, no previous systematic review has provided evidence for this association.

A recent systematic review conducted by Vancampfort in 2018 examined physical activity and suicidal ideation across the lifespan, which included adolescents (Vancampfort et al., 2018a). Compared to this systematic review, our research specifically targeted the adolescent population and specifically examined sports participation rather than physical activity, and, more importantly, three suicide-related outcomes (suicidal ideation, suicide plans, and suicide attempts) rather than only suicidal ideation. The most recent systematic review assessed the behavioral, psychological, and social impact of team sports on young athletes (Zuckerman et al., 2021). While Zuckerman et al. also investigated cross-sectional and longitudinal associations between team sports and suicidal ideation, it targeted young athletes under the age of 25, not only general or school-attending. In the 2018 systematic review, Vancampfort et al. found an unclear association between physical activity participation and suicidal ideation among adolescents (Vancampfort et al., 2018a), which is inconsistent with our findings. One possible explanation is that systematic review only included studies up to May 2017, whereas our research included the five most up-to-date articles since 2018. Therefore, compared to the previous study, there is more evidence to support exploring the association between sports participation and suicidal ideation among adolescents.

The most recent systematic review found that participation in team sports was associated with lower suicidal ideation among young athletes under 25 years of age, which is in line with our findings (Zuckerman et al., 2021). However, Zuckerman et al. only investigated the association between team sports participation and suicidal ideation among young athletes, whereas our research extended the suicide-related outcomes to suicide plans and suicide attempts. More importantly, the population that Zuckerman’s study investigated was young athletes under the age of 25 years. Previous epidemiological evidence suggests that as the incidence of suicide-related outcomes increases with age, but suicide is a more common cause of death in younger age groups (Bilsen, 2018). Evidence from the previous systematic review can only support the association between team sports participation and suicidal ideation in young athletes, while the results of our research can support associations between sports participation and the three suicide-related outcomes in a broader population of adolescents, thus guiding the direction of subsequent research and supporting policy formulation.

While the present study provides more evidence to support the association between sports participation and suicide-related outcomes among adolescents compared to previous systematic reviews. Among the total sample, only suicidal ideation and suicide attempts met the current definition of being consistently related to sports participation among adolescents. However, it is noteworthy that inconsistencies between studies were reported in the association between sports participation and suicide plans, and in the gender-stratified analysis of the association between sports participation and all three suicide-related outcomes. In terms of suicide plans, only four studies investigated the relationship between sports participation and suicide plans, and the sample size was not sufficient to support consistent results. In both gender-stratified analyses of suicidal ideation and suicide attempts, there were sufficient sample sizes to support the evidence. Nevertheless, in all comparisons, while the majority of variables reported associations with sports participation, few were significant. Consequently, there is difficulty in drawing consistent conclusions about these variables. The lack of consistency in the results may be caused by differences in measurements and samples, and confounding or moderating variables may need to be considered in the analyses.

The first possible explanation is the bias introduced by measurement, as measuring physical activity or sports participation in adolescents is a challenging task. While we have very accurate but invasive measures (e.g., double-labeled water), to more user-friendly device-based measures (e.g., accelerometers), to self-reported measures (e.g., questionnaires), they are all prone to bias and limitations (Baranowski et al., 1993). All the studies included in this systematic review employed unvalidated self-report instruments to measure adolescent sports participation, which may result in fewer significant associations being reported. Moreover, compared to physical activity or sedentary behavior, sports participation is more complicated and harder to accurately assess.

The second explanation is the targeted sample. This systematic review included observational studies of various designs, which led to a very large variation in the sample size of included studies, ranging from 739 to 152,858. In adolescents, the expected moderate correlation between sample size and proportion of significant associations may explain the inconsistent results that emerge (Sallis et al., 2000). Furthermore, cohort differences may have contributed to the inconsistent results. Even when two studies adopt the same study design, recruit the same participants, and use the same measures, it is possible to achieve the different results. This is because different cohorts have different demographic characteristics, such as various socio-economic status, educational attainment, gender ratio, and ethnic distribution. Although all the studies included in this systematic review were from high-income countries, which reduces cohort differences to some extent, it is still not possible to conduct subgroup analyses based on ethnicity or socio-economic status. There may be different correlations between boys’ and girls’ sports participation, and this study conducted subgroup analyses according to gender, but was incapable of drawing consistent conclusions.

Although this systematic review could not induce the consistent association between sports participation and several suicide-related outcomes, it reported an association between higher sports participation and less suicidal ideation and suicide attempts. The physiological and psychological mechanisms of human suicide are unclear (O'Connor and Nock, 2014), and the mechanisms of how sports participation reduces suicidal ideation, suicide plans, and suicide attempts are also uncertain (Brown, 2005). Some hypothetical mechanisms may explain the negative association between sports participation and these three suicide-related outcomes. Suicidal behavior is a complex process that begins with the emergence of suicidal ideation, moves on to planning the method, place and time frame for suicide, to making the attempt, and may even end in suicide death (Dumon and Portzky, 2014). The main finding in this present study was that sports participation was negatively associated with suicidal ideation and suicide attempts, while the association between sports participation and suicide plans was unclear. In the Stress-Diathesis Model of Suicidal Behavior developed by van Heeringen, suicidal behavior is hypothesized to be the result of an interaction between proximal stressors and distal personal vulnerabilities or qualities (van Heeringen, 2012). Previous studies have suggested that a healthy lifestyle, including physical activity and sports participation, can reduce adolescents’ vulnerability to stress and thus reduce suicidal thoughts, suicide plans, and suicide attempts (Brown and Lawton, 1986). Furthermore, vigorous physical activity and social support may be potential mediating pathways by which sports participation is associated with a reduced risk of suicidal ideation. Taliaferro et al. proposed that vigorous physical activity mediated the association between sports participation and self-esteem and depression, and these two risk factors for suicidal ideation moderated the association between vigorous physical activity and suicidal ideation, while there is also a mediating role for social support in sports participation and risk factors for suicidal ideation (Taliaferro et al., 2010). Previous studies using accelerometer-based measurements support a higher level of vigorous physical activity in adolescent sports participants than in non-sport participants (Pfeiffer et al., 2006; Telford et al., 2016). Therefore, sports participation may enable adolescents to engage in more vigorous physical activity, and participation in vigorous physical activity is associated with better mental health (Hrafnkelsdottir et al., 2018; Wassenaar et al., 2021), which can reduce suicidal ideation in adolescents. Another potential mediating factor is social support. Previous research has suggested that adolescents who participate in sports can maintain positive social relationships with activity participants, such as teammates, organizers, coaches, and families (Brown and Blanton, 2002; Taliaferro et al., 2011; Eime et al., 2013), which can lead to greater interpersonal and social support. Among adolescents, higher social support is associated with higher well-being (Chu et al., 2010), reduced risk for depression and anxiety (Rueger et al., 2016; Qi et al., 2020). Consequently, among adolescents, sports participation leads to better social support from activity participants, and better social support leads to better mental health, thus lowering the risk of suicide-related outcomes. Furthermore, physiological perspectives can be used to reveal potential mechanisms for sports participation in suicide prevention. For example, exercise can reduce sensitivity to stress and peripheral effects that affect metabolic function (Tsatsoulis and Fountoulakis, 2006), thus preventing negative effects caused by stress, including suicide-related outcomes (Gerber and Pühse, 2009). Furthermore, exercise-induced increases in serum β-endorphin concentrations are associated with altered euphoria and nociception, which can influence the psychological profile of adolescents and therefore reduce the risk of suicide (Harber and Sutton, 1984).

### 4.1 Limitation and further recommendation

This study is a systematic review conducted in strict compliance with the PRISMA guidelines (Page et al., 2021). Furthermore, this study used STROBE to assess the quality of the included studies and the best evidence synthesis consistent with previous studies (Vancampfort et al., 2012, 2018b). Thus, this study identified and evaluated all literature to date on sports participation and suicide-related outcomes among adolescents as scientifically as possible and drawn robust conclusions. Nevertheless, there are limitations to this present study. First, the cross-sectional studies included in this study were insufficient to support the directionality of the association between sports participation and suicide-related behaviors. While adolescents with low sports participation may experience suicide ideations, plans and attempts, adolescents who are prone to these suicide-related behaviors may not be prone to participate in sport. Therefore, more high-quality longitudinal studies are needed to determine the causal relationship between sports participation and suicide-related results. Second, there is heterogeneity in measuring sports participation and suicide-related outcomes, with measures of both exposure and outcome variables having unreported validity and reliability. This may introduce recall bias or social desirability bias and influence the corresponding synthesis of evidence. Therefore, there is a need to develop instruments with high reliability and validity in adolescents to assess their sports participation and suicide-related outcomes. Accelerometers or inclinometers can be adapted to estimate the different types of physical activity and sedentary behavior of young people during sports participation. Moreover, there are limitations to the included studies themselves. Few studies investigated suicide plans, and the sample sizes of the included studies varied excessively and there existed cohort differences. Finally, only papers published in English were included in this study.

## 5 Conclusion

This systematic review adds new evidence to support the association between higher level of sports participation and lower suicidal ideation and suicide attempts among adolescents. Whereas the association between sports participation and suicide plan is currently uncertain, and there is insufficient evidence to support differences between males and females.

## Data availability statement

The raw data supporting the conclusions of this article will be made available by the authors, without undue reservation.

## Author contributions

MH: Conceptualization, Formal analysis, Investigation, Methodology, Writing – original draft, Writing – review & editing, Data curation, Project administration. ZY: Data curation, Formal analysis, Software, Writing – original draft, Writing – review & editing. LY: Conceptualization, Methodology, Project administration, Supervision, Writing – review & editing. SC: Formal analysis, Methodology, Writing – original draft, Writing – review & editing.
